# Huaier restrains proliferative and invasive potential of human hepatoma SKHEP-1 cells partially through decreased Lamin B1 and elevated NOV

**DOI:** 10.1038/srep31298

**Published:** 2016-08-09

**Authors:** Zhongdong Hu, Ailin Yang, Guozhu Su, Yunfang Zhao, Ying Wang, Xingyun Chai, Pengfei Tu

**Affiliations:** 1 Modern Research Center for Traditional Chinese Medicine, Beijing University of Chinese Medicine, Beijing 100029, China; 2 School of Chinese Materia Medica, Beijing University of Chinese Medicine, Beijing 100102, China; 3 Department of Molecular Orthopaedics, Beijing Institute of Traumatology and Orthopaedics, Beijing Jishuitan Hospital, Beijing 100035, China

## Abstract

Hepatocellular carcinoma (HCC) is one of the most common cause of malignancy-related mortality worldwide. It is urgently needed to develop potential drugs with good efficacy and low toxicity for HCC treatment. The anti-tumor effect of Traditional Chinese Medicine (TCM) has received increasing attention worldwide. *Trametes robiniophila* Murr. (Huaier) has been used in TCM for approximately 1,600 years. Clinically, Huaier has satisfactory therapeutic effects in cancer treatment, especially in HCC. However, the mechanisms underlying the anti-cancer effect of Huaier remain ill defined. Herein we have demonstrated that Huaier dramatically inhibited cell proliferation and induced apoptosis in human hepatoma cell line SKHEP-1. Importantly, Huaier restrained the metastatic capability of SKHEP-1 cells. Mechanistically, down-regulation of Lamin B1 and up-regulation of Nephroblastoma overexpressed (NOV) were at least partially responsible for the inhibitory effect of Huaier on the proliferative and invasive capacity of SKHEP-1 cells. Our finding provided new insights into mechanisms of anti-HCC effect of Huaier and suggested a new scientific basis for clinical medication.

Hepatocellular carcinoma (HCC) is the fifth most frequent malignant tumors and the third leading cause of cancer-related deaths worldwide. HCC is also one of the most common cause of malignancy-related mortality in China^1^,^2^. Most HCC patients are usually diagnosed at an advanced stage generally with a poor prognosis^3^. Over the past 30 years, although the treatment of HCC has been improved, the prognosis of patients and the overall survival rate remain rather dismal^4^. So far, chemotherapy is the most common choice of treatment against the advanced HCC. However, the therapeutic effect of chemotherapeutic drugs is limited and discouraging because of some adverse effects including drug resistance and toxicity to normal cells^5^. Therefore, it is urgently needed to develop potential drugs with good efficacy and low toxicity for HCC treatment.

The anti-tumor effect of Traditional Chinese Medicine (TCM) has received increasing attention worldwide^6^,^7^. In China, *Trametes robiniophila* Murr. (Huaier) has been used in TCM for approximately 1,600 years. Many clinical applications have shown that Huaier has satisfactory therapeutic effects in the treatment of solid malignancies including liver cancer, gastric cancer, colon cancer, breast cancer, and lung cancer^8^,^9^. Huaier has no obvious toxicity and can be used alone or combined with other drugs. Huaier is able to improve the organic immunity, induce apoptosis of cancer cells, and inhibit angiogenesis^9^,^10^,^11^,^12^. However, the mechanisms underlying the anti-cancer effect of Huaier remain elusive.

In this study, the anti-tumor effect of Huaier in human hepatoma cell line SKHEP-1 was investigated. We have demonstrated that Huaier dramatically inhibited proliferation of SKHEP-1 cells. Moreover, Huaier treatment induced apoptosis in SKHEP-1 cells. Importantly, Huaier restrained the metastatic capability of SKHEP-1 cells. Mechanistically, we found that Huaier downregulated Lamin B1 expression and upregulated NOV expression in SKHEP-1 cells.

## Results

### Huaier inhibited proliferation of SKHEP-1 cells

**Figure 1:**
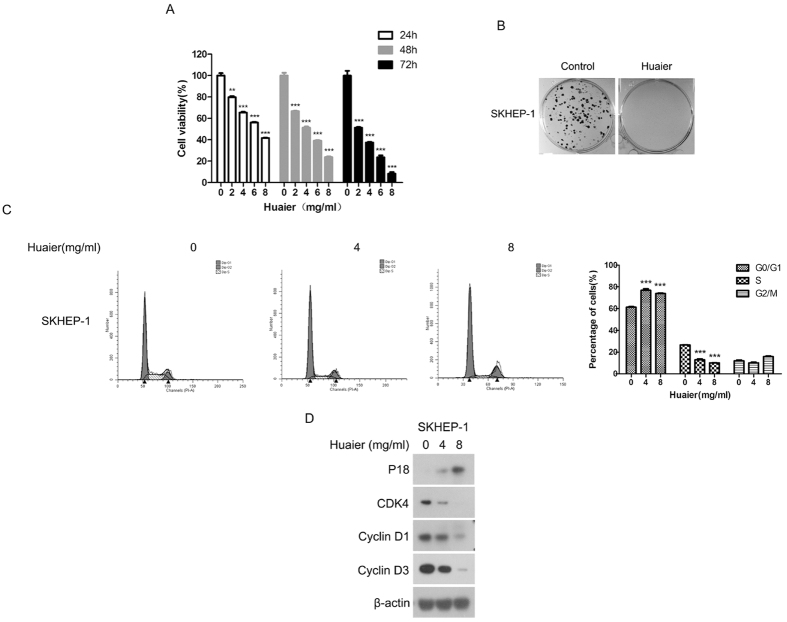
Huaier inhibited proliferation of SKHEP-1 cells. (**A**) SKHEP-1 cells treated with Huaier aqueous extract at the concentrations of 0, 2, 4, 6, 8 mg/ml for 24 h, 48 h, and 72 h, respectively, were subjected to cell viability assay. **P < 0.01, ***P < 0.001. (**B**) SKHEP-1 cells treated with or without Huaier aqueous extract (2 mg/ml) for 10 days were stained with crystal violet. Representative images were presented. (**C**) Cell cycle distribution of SKHEP-1 cells treated with Huaier aqueous extract at the concentrations of 0, 4, 8 mg/ml for 48 h were evaluated by flow cytometry. ***P < 0.001. (**D**) SKHEP-1 cells treated with Huaier aqueous extract at the concentrations of 0, 4, 8 mg/ml for 48 h were subjected to immunoblotting for indicated proteins.

To examine the effect of Huaier aqueous extract on proliferation of SKHEP-1 cells, we measured cell viability of SKHEP-1 cells treated with Huaier aqueous extract at the indicated concentrations (0, 2, 4, 6, and 8 mg/ml) for 24 h, 48 h, and 72 h, respectively. Our result indicated that Huaier aqueous extract significantly inhibited proliferation of SKHEP-1 cells (Fig. 1A). Moreover, colony formation assay was performed to evaluate the effect of Huaier aqueous extract on SKHEP-1 cell proliferation. As shown in Fig. 1B, Huaier treatment dramatically suppressed the formation of colonies derived from SKHEP-1 cells. Next, to explore whether cell-cycle arrest was responsible for the inhibitory effect of Huaier aqueous extract on proliferation of SKHEP-1 cells, flow cytometry assay was performed to examine cell-cycle distribution of SKHEP-1 cells treated with Huaier. As depicted in Fig. 1C, SKHEP-1 cells exposed to Huaier exhibited remarkably increased fraction of the G0/G1 phase and reduced fraction of the S phase, indicating that Huaier induced cell-cycle arrest in G0/G1 phase in SKHEP-1 cells. To clarify the mechanisms of cell-cycle arrest in G0/G1 phase induced by Huaier in SKHEP-1 cells, we examined the expression of cell cycle regulatory molecules. As shown in Fig. 1D, Huaier treatment enhanced the expression of CDK inhibitor p18 and reduced the expression of CDK4, Cyclin D1, and Cyclin D3 in a dose-dependent manner. Collectively, these data revealed that Huaier-induced G0/G1 phase arrest via p18 pathway partially contributes to inhibition of SKHEP-1 cell proliferation.

### Huaier induced apoptosis in SKHEP-1 cells

**Figure 2:**
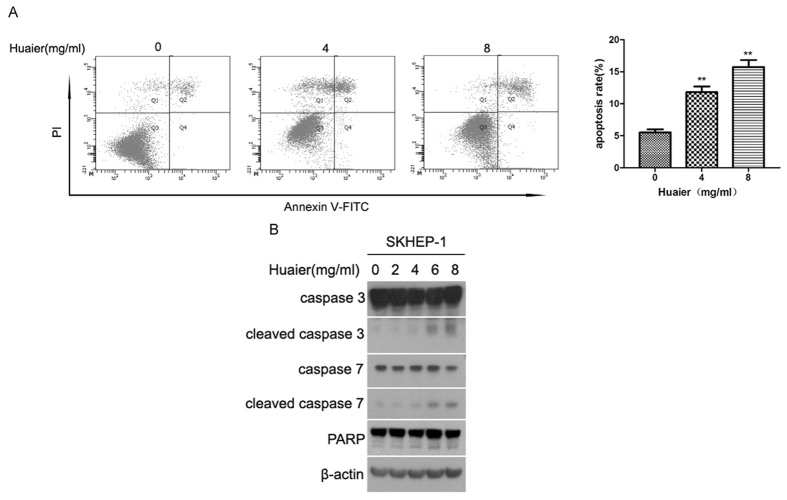
Huaier induced apoptosis in SKHEP-1 cells. (**A**) SKHEP-1 cells treated with or without Huaier aqueous extract for 48 h were subjected to apoptosis analysis by flow cytometry. Cells in the Q4 and Q2 quadrants were respectively regarded as early apoptotic and late apoptotic, and total cells in Q4 + Q2 quadrants were regarded as apoptotic. **P < 0.01. (**B**) SKHEP-1 cells treated with Huaier aqueous extract at the concentrations of 0, 2, 4, 6, 8 mg/ml for 48 h were subjected to immunoblotting for indicated proteins.

To determine the effect of Huaier aqueous extract on apoptosis in SKHEP-1 cells, we performed flow cytometry analysis with Annexin V-FITC/propidium iodide (PI) staining. As shown in Fig. 2A, the apoptosis rate of SKHEP-1 cells exposed to Huaier increased in a dose-dependent manner. To investigate whether the apoptosis induced by Huaier was attributed to caspase activation, we harvested total cell lysates after treatment and performed immunoblotting analysis. caspase-3 and caspase-7 are both critical executioner of apoptosis^13^,^14^. Cleavage of PARP, a substrate of caspase-3 and caspase-7, serves as a marker of apoptosis^15^,^16^. As shown in Fig. 2B, cleavage of caspase-3, caspase-7, and PARP were increased after treatment with Huaier in a dose-dependent manner in SKHEP-1 cells, suggesting that activation of caspase-3 and caspase-7 mediate Huaier-induced apoptosis. Taken together, Huaier aqueous extract promotes apoptosis of SKHEP-1 cells.

### Huaier inhibited metastatic capability of SKHEP-1 cells

**Figure 3:**
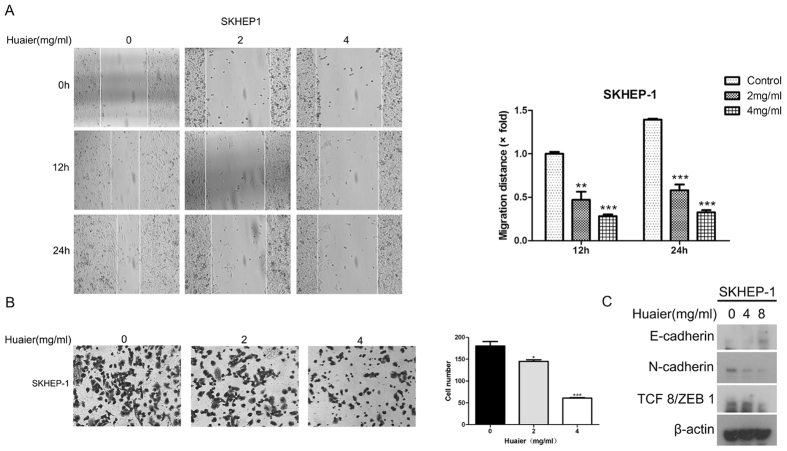
Huaier inhibited metastatic capability of SKHEP-1 cells. (**A**) SKHEP-1 cells treated with Huaier aqueous extract at the concentrations of 0, 2, 4 mg/ml were subjected to wound healing assay and observed at 0 h, 12 h, and 24 h, respectively. (Left panel): Representative images (100×); (Right panel): quantitative data. **P < 0.01, ***P < 0.001. (**B**) SKHEP-1 cells treated with Huaier aqueous extract at the concentrations of 0, 2, 4 mg/ml for 24 h were subjected to Transwell assay. (Left panel): Representative images (200×); (Right panel): quantitative data. (**C**) SKHEP-1 cells treated with or without Huaier aqueous extract for 48 h were subjected to immunoblotting for indicated proteins.

Tumor cell migration and invasion play vital roles in cancer metastasis^17^,^18^. It has been reported that SKHEP-1 cells exhibit highly metastatic capacity^19^. To evaluate the migration capacity of SKHEP-1 cells *in vitro*, we performed wound healing assay. As depicted in Fig. 3A, the migration distance of SKHEP-1 cells were reduced by Huaier in a dose-dependent manner, indicating that Huaier significantly inhibits migration ability of SKHEP-1 cells. Moreover, to investigate the invasive capacity of SKHEP-1 cells *in vitro*, Transwell assay was performed in SKHEP-1 cells exposed to Huaier aqueous extract. Result from Transwell assay revealed that the number of successfully invading SKHEP-1 cells was significantly decreased (Fig. 3B), which showed that treatment with Huaier suppressed the invasive capacity of SKHEP-1 cells. The epithelial-mesenchymal transition (EMT) exerts an important role in cancer metastasis^20^,^21^. EMT is characterized by the loss of epithelial markers and the gain of mesenchymal markers^22^. Next, we explored the effect of Huaier on EMT of SKHEP-1 cells. As shown in Fig. 3C, Huaier treatment upregulated the expression of epithelial marker, E-cadherin, and downregulated the expression of mesenchymal markers, N-cadherin and TCF8/ZEB1^22^, in SKHEP-1 cells, indicating that Huaier could reverse EMT in SKHEP-1 cells. Collectively, Huaier suppressed metastasis of SKHEP-1 cells *in vitro*.

### Huaier induced the down-regulation of Lamin B1 and up-regulation of NOV in SKHEP-1 cells

**Figure 4:**
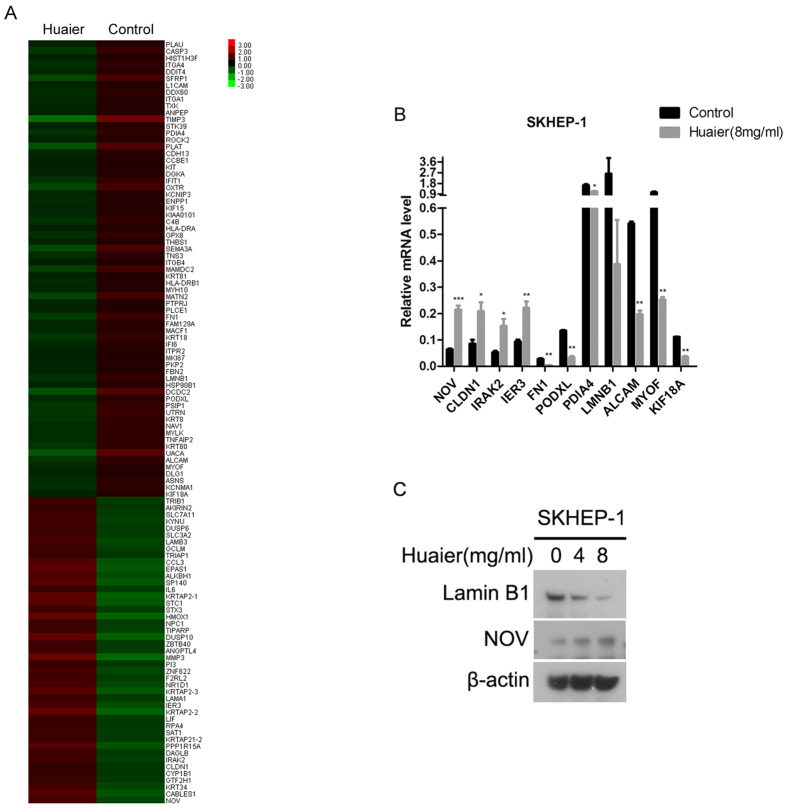
Huaier downregulates Lamin B1 and upregulates NOV in SKHEP-1 cells. (**A**) Hierarchical clustering of cancer-related genes differentially expressed in SKHEP-1 cells treated with or without Huaier aqueous extract (8 mg/ml) for 48 h. Red represents high expression and green represents low expression. (**B**) Total RNA extracted from SKHEP-1 cells treated with or without Huaier aqueous extract (8 mg/ml) for 48 h was subjected to Quantitative real-time PCR. *P < 0.05, **P < 0.01, ***P < 0.001. (**C**) SKHEP-1 cells treated with or without Huaier aqueous extract for 48 h were subjected to immunoblotting for indicated proteins.

To investigate the molecular mechanisms of anti-tumor effect of Huaier, we performed gene expression profiling analysis on SKHEP-1 cells treated with or without Huaier extract. Upon Huaier treatment, there were 730 genes of more than 2-fold change in expression, of which 362 were upregulated genes and 368 were downregulated genes. As shown in Fig. 4A, some cancer-related genes expression showed distinct changes after Huaier treatment. Through literature investigation on the functions of the differentially expressed genes, several candidate genes were selected for the validation by real-time PCR. Eventually, 11 candidate genes (*LMNB1*, *ALCAM*, *MYOF*, *KIF18A*, *FN1*, *PODXL*, *PDIA4*, *NOV*, *CLDN1*, *IRAK2*, and *IER3*) had corresponding changes after Huaier treatment (Fig. 4B). Next, two candidate genes, *LMNB1* and *NOV* reported to be pro- and anti-oncogenic^23^,^24^,^25^,^26^, respectively, were chosen for further confirmation by immunoblotting analysis. As shown in Fig. 4C, the expression of Lamin B1 protein encoded by the *LMNB1* gene was reduced whereas NOV expression was increased in a dose-dependent manner in response to Huaier, which was consistent with the results obtained by microarray analysis and by real-time PCR.

### Effects of Lamin B1 and NOV on proliferation and metastasis of SKHEP-1 cells

**Figure 5:**
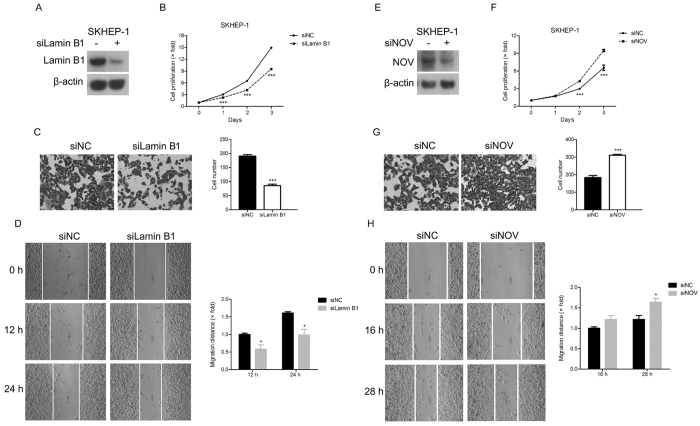
Effects of Lamin B1 and NOV on proliferation and metastasis of SKHEP-1 cells. (**A**) SKHEP-1 cells were transfected with Lamin B1 siRNAs for 48 h and then subjected to immunoblotting for Lamin B1. (**B**) The proliferation of SKHEP-1 cells transfected with negative control siRNAs or Lamin B1 siRNAs was examined by CCK-8 assay. (**C**) SKHEP-1 cells transfected with negative control siRNAs or Lamin B1 siRNAs were subjected to Transwell assay. (Left panel): Representative images (200×); (Right panel): quantitative data. (**D**) SKHEP-1 cells transfected with negative control siRNAs or Lamin B1 siRNAs were subjected to wound healing assay and observed at 0 h, 12 h, and 24 h, respectively. (Left panel): Representative images (100×); (Right panel): quantitative data. (**E**) SKHEP-1 cells were transfected with NOV siRNAs for 48 h and then subjected to immunoblotting for NOV. (**F**) The proliferation of SKHEP-1 cells transfected with negative control siRNAs or NOV siRNAs was examined by CCK-8 assay. (**G**) SKHEP-1 cells transfected with negative control siRNAs or NOV siRNAs were subjected to Transwell assay. (Left panel): Representative images (200×); (Right panel): quantitative data. (**H**) SKHEP-1 cells transfected with negative control siRNAs or NOV siRNAs were subjected to wound healing assay and observed at 0 h, 16 h, and 28 h, respectively. (Left panel): Representative images (100×); (Right panel): quantitative data. *P < 0.05, ***P < 0.001.

To explore whether the decreased Lamin B1 and increased NOV induced by Huaier were responsible for the suppression of proliferation and metastasis in SKHEP-1 cells exposed to Huaier, we investigated the effects of blockage of Lamin B1 expression or NOV expression on proliferation and metastasis of SKHEP-1 cells by using RNA interference. As shown in Fig. 5A, Lamin B1 expression was indeed dramatically impaired in SKHEP-1 cells with siRNAs targeting Lamin B1 gene. Reduction of Lamin B1 expression significantly blunted the proliferation of SKHEP-1 cells (Fig. 5B). Moreover, SKHEP-1 cells transfected with Lamin B1 siRNAs exerted decreased invasive and migratory capacity (Fig. 5C,D). Additionally, we also knocked down NOV expression with siRNAs in SKHEP-1 cells (Fig. 5E). Interestingly, the depletion of NOV expression accelerated the proliferation of SKHEP-1 cells (Fig. 5F). Furthermore, SKHEP-1 cells transfected with NOV siRNAs had enhanced invasive and migratory capacity (Fig. 5G,H). Collectively, we proposed that the down-regulation of Lamin B1 and up-regulation of NOV at least partially contributed to the inhibitory proliferation and metastasis of SKHEP-1 cells in response to Huaier treatment.

## Discussion

TCM has been extensively applied for cancer treatment in China. Recently, increasing attentions have been paid to anti-tumor drugs originating from TCM^7^. A lot of clinical applications have revealed that Huaier has a good anti-tumor activity, especially in HCC^27^,^28^,^29^. Nevertheless, the underlying mechanisms of anti-HCC effect of Huaier remain ill defined. In this study, we have demonstrated that suppression of proliferation and metastasis, and induction of apoptosis in SKHEP-1 cells by Huaier were at least partially due to down-regulation of Lamin B1 and up-regulation NOV.

Dysregulation of cellular proliferation and apoptosis are critical characteristics of cancers. Thus, the modulation of proliferation and apoptosis in cancer cells is a rational and feasible strategy for cancer treatment. In this study, we demonstrated that Huaier significantly inhibited the proliferation of human hepatoma cell line SKHEP-1, which was partially due to Huaier-induced cell-cycle arrest in G0/G1 phase (Fig. 1). Our result was consistent with a previous finding that Huaier suppressed cell viability and induced G0/G1 cell-cycle arrest in breast cancer cell lines^12^. However, there are several studies reporting that Huaier induced cell-cycle arrest in G2/M phase in human cervical cancer cells^30^ and human melanoma cells^31^, and caused S phase arrest in human hepatocellular carcinoma cell lines HepG2 and Bel-7402^28^. This phenomenon may be interpreted by the complicated effects of Huaier on different cell lines. We illuminated that Huaier-induced apoptosis was mediated by activation of caspase-3, caspase-7, and PARP in SKHEP-1 cells (Fig. 2), which was consistent with previous studies revealing that Huaier could induce caspase 3-mediated apoptosis in human melanoma cells^31^, human breast cancer cells^12^, and human hepatocellular carcinoma cells^28^.

High metastatic potential of HCC is mainly responsible for the poor prognosis and high mortality of patients^32^,^33^. Thus, the blocking of metastasis is an effective approach for HCC therapy. Consequently, the development of new drugs that inhibit cancer metastasis with lower toxicity is extremely urgent. Previous studies uncovered that Huaier repressed metastasis in human breast cancer cell lines^12^ as well as in human ovarian cancer cell lines^34^. Given that SKHEP-1 is a human hepatoma cell line with highly metastatic capacity, we chose SKHEP-1 cells as a cell model for investigating the effect of Huaier on HCC metastasis. In this study, we demonstrated that Huaier treatment significantly inhibits migration ability and invasive capacity of SKHEP-1 cells (Fig. 3A,B). Additionally, Huaier resisted the EMT process of SKHEP-1 cells (Fig. 3C). Therefore, Huaier exerts good anti-metastatic potential in SKHEP-1 cells *in vitro*. Huaier may serve as a candidate drug targeting on metastasis in HCC therapy.

The underlying mechanisms of anti-tumor effect of Huaier remain less well characterized. AKT/GSK3β/β-catenin signaling, ERα signaling, and JNK/p38 signaling were identified to participate in the inhibition of proliferation and metastasis of human cancer cells by Huaier^30^,^34^,^35^. Lamin B1 is one of the lamin family members which are highly conserved proteins making up nuclear matrix^36^. Lamin B1 is aberrantly overexpressed in human pancreatic cancer, prostate cancer, and hepatocellular carcinoma^23^,^24^,^37^. Depletion of Lamin B1 inhibited the proliferation and invasion of pancreatic cancer cells^23^. In addition, NOV is a matricellular protein from the CCN family and exerts tumor-suppressive activities in various cancers^25^,^26^,^38^. In the current work, Lamin B1 and NOV were screened out through gene expression profiling analysis. Moreover, quantitative real-time PCR and immunoblot analysis confirmed that Huaier induced the down-regulation of Lamin B1 and up-regulation of NOV in SKHEP-1 cells (Fig. 4). Furthermore, Lamin B1 and NOV were respectively identified as positive and negative regulators of proliferation and metastasis of SKHEP-1 cells (Fig. 5). Taken together, Huaier inhibited proliferation and cell motility of SKHEP-1 cells, in part due to decreased Lamin B1 and increased NOV.

In summary, this study identified two novel targets and provided new insight into mechanisms underlying anti-HCC effect of Huaier, revealing that Huaier restrained proliferation and cell motility of human hepatoma cell line SKHEP-1 partially via regulation of Lamin B1/NOV.

## Materials and Methods

### Reagents and antibodies

DMEM, fetal bovine serum, penicillin-streptomycin solution, and 0.25% trypsin were obtained from Corning; DMSO (sigma); Cell Counting Kit-8 (CCK-8) from Dojindo; Annexin V-FITC apoptosis detection kit (BD Pharmingen™). β-actin was purchased from Abgent. Cyclin D1, Cyclin D3, CDK4, p18, caspase-3, caspase-7, PARP, E-cadherin, N-cadherin, and TCF8/ZEB1 antibodies were purchased from Cell Signaling Technology. Lamin B1, NOV, and the HRP-labeled anti-mouse and anti-rabbit antibodies were from Santa Cruz Biotechnology.

### Preparation of Huaier extract

The electuary ointment of Huaier was obtained from Gaitianli Medicine Co. Ltd (Jiangsu, China) and dissolved in complete medium adequately to obtain the 10 mg/ml stock solution, which was then filtrated with a 0.22 μm filter and stored at 4 °C for short-term use. As shown in Supplementary Fig. S1, HPLC chromatogram (A) suggested that Huaier extract contain ingredients with strong and moderate polarity, almost no lipophilic constituents. But, there were obvious peaks observed in LC-IT-TOF-MS positive mode (B) chromatogram. The LC-MS analysis suggested that major components (retention time at 0–10 min) of Huaier are of strong polarity.

### Cell culture

Human hepatoma cell line SKHEP-1 is from American Type Culture Collection. SKHEP-1 cells were cultured in DMEM with 10% fetal bovine serum and 1% penicillin-streptomycin at 37 °C and 5% CO_2_.

### Cell viability assay

Cells were seeded in a 96-well plate at a density of 3,000 cells/well, and then cells were treated with Huaier extract at the indicated concentrations for 24 h, 48 h, and 72 h, respectively. 10 μl of CCK-8 was added into the 96-well plate. After 2 h of incubation, the optical density was examined with a microplate reader at 450 nm.

### Colony formation

SKHEP-1 cells were seeded in six-well plate at a density of 1000 cells/well. The next day, cells were treated with Huaier aqueous extract. 10 days after treatment, cells were fixed with methanol for 15 min and then stained with 0.1% crystal violet. At last, cell colonies stained were photographed with a digital camera.

### Apoptosis detection

SKHEP-1 cells were seeded in a six-well plate at a density of 1 × 10^5^ cells/well and then treated with Huaier extract for 48 h. The cells were collected for apoptosis analysis using an Annexin V-FITC apoptosis detection kit (BD Pharmingen™) according to the manufacturer’s protocol. Annexin V-FITC positive and PI negative cells were considered as early apoptotic, whereas Annexin V-FITC positive and PI positive cells were considered as late apoptotic.

### Cell cycle analysis

SKHEP-1 cells were seeded in six-well plate. The next day, cells were starved in serum-free medium for 12 h, and then cells were treated with Huaier aqueous extract at the concentrations of 0, 4, 8 mg/mL for 48 h. Cells were collected for cell cycle analysis using a cell cycle and apoptosis analysis kit (Biyuntian Biotech Co., Ltd, Shanghai, China) according to the manufacturer’s protocol.

### Wound healing assay

SKHEP-1 cells were seeded in 12-well plates. As the cells grew up to a subconfluent state, the culture medium was replaced with serum-free medium. After 12 h, a straight cell-free wound was scratched with a 10 μl pipette tip. And then cells were washed twice with PBS and maintained in serum-free medium containing Huaier aqueous extract. The cell scratches were observed and measured at 0 h, 12 h, and 24 h. The cell migration distances were analyzed quantitatively.

### Transwell assay

The Matrigel (B.D. Biosciences Pharmingen) was dissolved in DMEM at a ratio of 1:12. 60 μl mixture was added to the upper chamber of Transwell gently. Transwell chamber was placed in 24-well plate to set up the transwell system. And then the transwell system was maintained at 37 °C for 4 h. SKHEP-1 cells were starved in serum-free medium for 12 h. 1 × 10^5^ cells were collected and resuspended in 200 μl serum-free medium containing different concentrations of Huaier aqueous extract, and then added to upper chamber. 750 μl complete medium with 10% FBS was added to 24-well plate. After incubation at 37 °C for 24 h, the cells on the upper side of the membrane were wiped off with a cotton swab. The cells on the bottom surface of the membrane were fixed with methanol for 15 min and then stained with crystal violet for 10 min. The invading cells were photographed on 5 random fields under an inverted microscope.

### Quantitative real-time PCR

Total RNA was extracted from SKHEP-1 cells treated with or without Huaier aqueous extract (8 mg/ml) for 48 h with E.Z.N.A.^®^ Total RNA Kit I (OMEGA), and then converted into cDNA using the PrimeScript RT Reagent Kit (TaKaRa) according to the manufacturer instructions. 4 μl of cDNA was used for the quantitative PCR by using the TransStart Top Green qPCR SuperMix (TransGen Biotech). The primer sequences were as follows:

Human NOV forward: CTTGGTGCGGAGACACTTTT,

Human NOV reverse: ATGCTGAAACAGACTCGGCT;

Human LMNB1 forward: TTCTCGAAGCTTGATCTGGG,

Human LMNB1 reverse: GATCGAGCTGGGCAAGTG;

Human FN1 forward: CGGTGGCTGTCAGTCAAAG,

Human FN1 reverse: AAACCTCGGCTTCCTCCATAA;

Human IER3 forward: TGGTGAGCAGCAGAAAGAGA,

Human IER3 reverse: CGCAGGGTTCTCTACCCTC;

Human IRAK2 forward: TGGAATGGGACACCTGATTT,

Human IRAK2 reverse: GCAACTTGTGGACCTCCTGT;

Human CLDN1 forward: TCACTCCCAGGAGGATGC,

Human CLDN1 reverse: GGCAGATCCAGTGCAAAGTC;

Human PDIA4 forward: GGCAGGCTGTAGACTACGAG,

Human PDIA4 reverse: TTGGTCAACACAAGCGTGACT;

Human PODXL forward: TGTTTTGTTAGATGAGTCCGTAGTA,

Human PODXL reverse: CGCTGCTGCTACTGTTGTCA;

Human ALCAM forward: AGGTACGTCAAGTCGGCAAG,

Human ALCAM reverse: CTTCTGCCTCTTGATCTCCG;

Human MYOF forward: TAATTGGCACGGCGACTGTAG,

Human MYOF reverse: GGAGATCAGCTTGTACGGCAG;

Human KIF18A forward: CTTTCAAGGGAGATGGCATT,

Human KIF18A reverse: GGACCAGTTCAGCCTATTCCT;

Human β-actin forward: GTTGTCGACGACGAGCG,

Human β-actin reverse: GCACAGAGCCTCGCCTT;

### Immunoblotting

Cells were treated with Huaier extract at the indicated concentrations for 48 h, and then washed 2 times with ice-cold PBS and harvested with lysis buffer (10 mM Tris (pH 6.8), 2% SDS, 10% glycerol, 100 mM DTT). The protein levels were examined by western blotting described previously^39^.

### RNA interference

The siRNA oligonucleotides were synthesized from GenePharma (Shanghai, China). SKHEP-1 cells seeded in 6-well plates were transfected with siRNAs against Lamin B1 or NOV with Lipofectamine 2000 according to the manufacturer’s instructions. The siRNA target sequences were as follows: Negative Control (NC): 5′-TTCTCCGAACGTGTCACGT-3′; Lamin B1(human): 5′-CGCGCTTGGTAGAGGTGGA-3′; NOV(human): 5′-CACACCAACTGTCCTAAGA-3′.

### Transcriptome microarray analysis

RNA extracted from SKHEP-1 cells treated with or without Huaier extract were subjected to microarray on Affymetrix GeneChip 645 System with Affymetrix GeneChip Human Transcriptome Array (HTA) 2.0. The data were analyzed using the Affymetrix Expression console software by Beijing Auwigene Tech. Ltd.

### Statistical analysis

Data represents the mean ± SEM of triplicate samples. Differences between two groups were analyzed with the 2-tailed Student *t*-test in GraphPad Prism 5.0 software. It was considered to be statistically significant when *P* < 0.05.

## Additional Information

**How to cite this article**: Hu, Z. *et al*. Huaier restrains proliferative and invasive potential of human hepatoma SKHEP-1 cells partially through decreased Lamin B1 and elevated NOV. *Sci. Rep*. **6**, 31298; doi: 10.1038/srep31298 (2016).

## Supplementary Material

Supplementary Information
