# Supplementary material for: Huaier restrains proliferative and invasive potential of human hepatoma SKHEP-1 cells partially through decreased Lamin B1 and elevated NOV
Source: Sci Rep. 2016 Aug 9;6:31298. doi: 10.1038/srep31298 (PMC4977525; doi:10.1038/srep31298)
Supplement: Supplementary Information [file srep31298-s1.pdf]

# Huaier restrains proliferative and invasive potential of human hepatoma SKHEP-1 cells partially through decreased Lamin B1 and elevated NOV

Zhongdong Hu[1,†], Ailin Yang[1,2,†], Guozhu Su[1,2], Yunfang Zhao[1], Ying Wang[3], Xingyun Chai[1], and Pengfei Tu[1*]

[1]Modern Research Center for Traditional Chinese Medicine, Beijing University of Chinese Medicine, Beijing 100029, China.

[2]School of Chinese Materia Medica, Beijing University of Chinese Medicine, Beijing 100102, China.

[3]Department of Molecular Orthopaedics, Beijing Institute of Traumatology and Orthopaedics, Beijing Jishuitan Hospital, Beijing 100035, China

[*]Corresponding Author: Pengfei Tu, North Third Ring Road No. 11, Chaoyang District, Beijing 100029, P. R. China, tel/fax: 8610 8280 2750, e-mail: pengfeitu@163.com.

[†]These authors contributed equally to this work.

## Supplementary Methods

*Instrument, reagents and LC-DAD-IT-TOF-MS analysis*

Liquid chromatography was conducted on a Shimadzu LC system (Shimadzu, Kyoto, Japan) consisting of two LC-20AD$_{XR}$ solvent delivery units, a SIL-20AC$_{XR}$ autosampler, a CTO-20AC column oven, a SPD-M20A diode array detection (DAD) module, a DGU-20A$_{3R}$ degasser, and a CBM-20A controller. A hybrid ion trap-time-of-flight mass spectrometer (Shimadzu) equipped with an electrospray ionization (ESI) source was connected to the LC system *via* a PEEK tube (0.13 mm i.d.) to carry out high-resolution tandem mass spectrometry. Chromatographic separation was conducted on a Zorbax SB C18 column (250 × 4.6 mm, 5 µm, Agilent) at a flow rate of 1.0 mL/min, Methanol (M)–0.1% aqueous with formic acid (B) and with a gradient program as follows: 0–50 min, 5–95% M; 50–60 min, 95% M. Roughly, 20% portion of the effluent was introduced into the ESI interface by splitting the effluent *via* two PEEK tubes with a length ratio of 1:4. At the end of each run, the initial composition of mobile phase (5% M) was delivered to re-equilibrate the whole system for 10 min. The injection volume was set at 8 µL, UV absorption over 190–400 nm was recorded by DAD unit and a fixed wavelength of 265 nm.

The optimized operating conditions of HRMS analysis were as follows: positive mode; nebulizer gas ($N_2$) flow, 1.5 L/min; curved desolvation line (CDL) temperature, 200 °C; heat block temperature, 200 °C; detector voltage, 1.40 kV; interface voltage (+), 4.5 kV; ion accumulated time, 10 ms; repeat times, 2; collision energy was set at 50% for $MS^2$; pressure of ion trap, 1.9e-002 Pa; pressure of TOF region, 1.2e-004 Pa; scan range *m/z* 100–1000; precursor ion isolation, 3.0000 Da. An automatic scan mode was used in the HPLC-ESI-$MS^n$ analysis.

Methanol and formic acid of LC-MS grade were product of Fisher Scientific

(FairLawn, NJ, USA). Ultrapure water was prepared in our own laboratory by Milli-Q plus System (Millipore, Bedford, MA, USA). Analytical-grade solvents used for sample preparation are products of Beijing Chemical Factory (Beijing, China).

**Supplementary Figure**

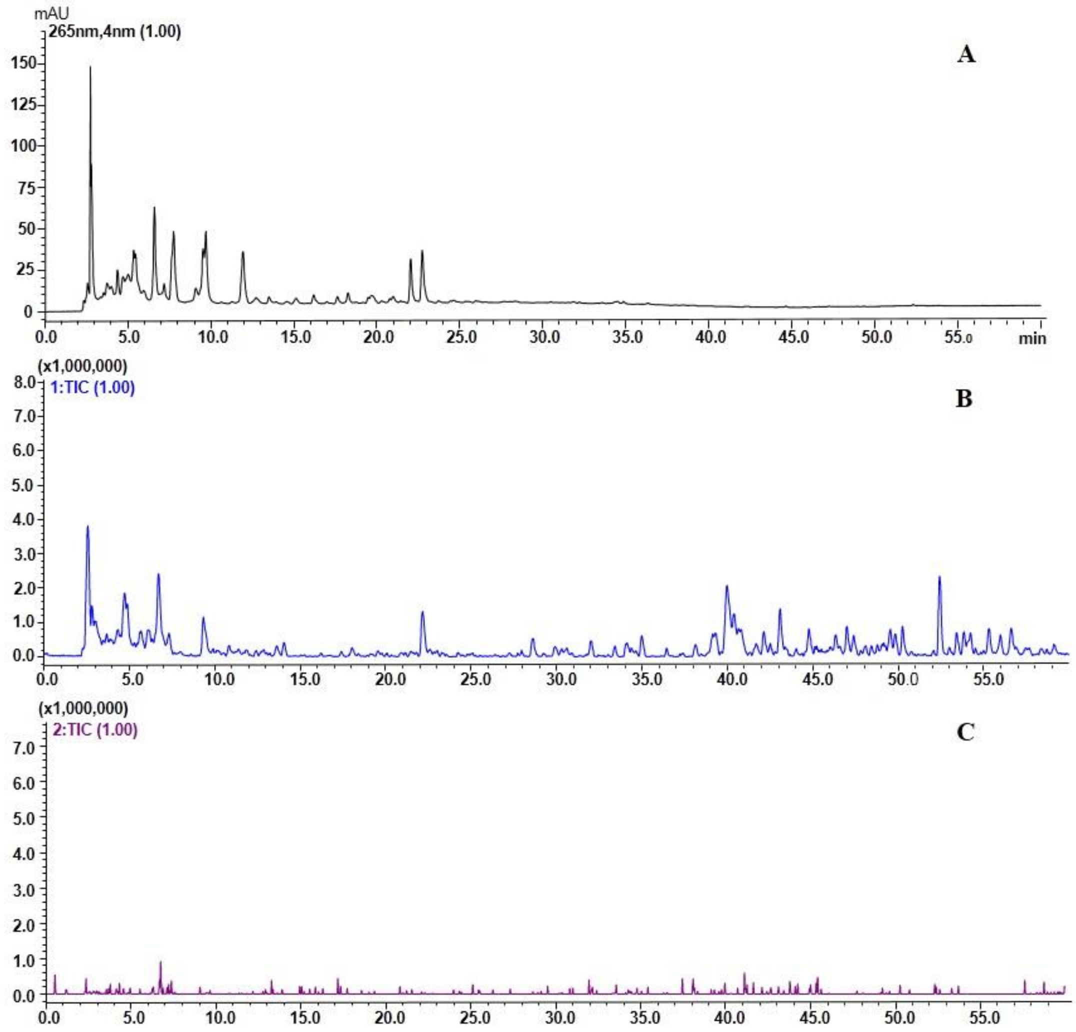


Figure S1. HPLC-DAD (A), LC-IT-TOF-MS in positive mode (B) and negative mode (C) chromatograms of Huaier extract. The chromatographic separation was performed on a Zorbax SB C18 column (250 × 4.6 mm, 5 μm, Agilent). Methanol (M)-0.1% aqueous with formic acid (B) were used as the mobile phase for LC-DAD-IT-TOF-MS analysis. The flow rate was set at 1.0 mL/min and the detection wavelength was set at 265 nm. The elution condition was applied with a gradient program as follows: 0–50 min, 5%–95% M; 50–60 min, 95% M. Aliquots of 8 μL were injected into LC-DAD-IT-TOF-MS system for analysis.
